# Old and New Adjunctive Therapies in Celiac Disease and Refractory Celiac Disease: A Review

**DOI:** 10.3390/ijms241612800

**Published:** 2023-08-15

**Authors:** Marco Valvano, Stefano Fabiani, Sabrina Monaco, Mauro Calabrò, Antonio Mancusi, Sara Frassino, Claudia Rolandi, Marta Mosca, Susanna Faenza, Emanuele Sgamma, Nicola Cesaro, Giovanni Latella

**Affiliations:** Gastroenterology Unit, Division of Gastroenterology, Hepatology and Nutrition, Department of Life, Health and Environmental Sciences, University of L’Aquila, Piazzale Salvatore Tommasi 1, 67100 L’Aquila, Italy; marco.valvano@graduate.univaq.it (M.V.); stefano.fabiani1@graduate.univaq.it (S.F.); sabrina.monaco@graduate.univaq.it (S.M.); mauro.calabro@graduate.univaq.it (M.C.); antonio.mancusi@graduate.univaq.it (A.M.); sara.frassino@graduate.univaq.it (S.F.); claudia.rolandi@graduate.univaq.it (C.R.); marta.mosca@graduate.univaq.it (M.M.); susanna.faenza@graduate.univaq.it (S.F.); emanuelesgamma94@gmail.com (E.S.); nicola.cesaro@graduate.univaq.it (N.C.)

**Keywords:** celiac disease, refractory celiac disease, corticosteroid, mesalamine, small molecules

## Abstract

Celiac disease (CD) is a chronic enteropathy caused by the ingestion of gluten in a genetically susceptible individual. Currently, a gluten-free diet (GFD) is the only recommended treatment. However, unintentional gluten ingestion or a persistent villous atrophy with malabsorption (regardless of a strict GFD) as in the case of Refractory Celiac Disease (RCD) represents a major issue. In this review, we have analysed and discussed data from both randomized controlled trials and observational studies concerning adjunctive therapies as well as novel therapies for the treatment of CD and RCD. The literature search was carried out through Medline and Scopus. In total, 2268 articles have been identified and 49 were included in this review (36 studies resulting from the search strategy and 13 from other sources). Today, GFD remains the only effective treatment, although steroids, mesalamine, and more recently biological therapies have found space in the complex management of RCD. Currently, studies evaluating the effectiveness of novel therapies are still limited and preliminary results have been controversial.

## 1. Introduction

Celiac disease (CD) is a chronic autoimmune disease caused by the ingestion of gluten in genetically susceptible individuals that mainly affects the small bowel [1]. It is one of the most common food-related chronic conditions, with a worldwide prevalence rate of 1.4%, thus becoming a considerable global public health concern [2]. The disease could appear with intestinal symptoms (diarrhoea, abdominal pain), or less frequently with extra-intestinal symptoms, such as anaemia, osteoporosis, and neuropathy, or it could be asymptomatic [3]. Gluten-Free Diet (GFD) remains the only recommended treatment for celiac disease and usually results in improvement or resolution of enteropathy and symptoms [3]. Nevertheless, diet itself can cause nutritional deficiencies that need to be periodically checked, such as vitamin B12, folate, and vitamin D deficiency, or constipation due to reduced fibre intake [4,5]. Moreover, many processed gluten-free products have an increased glycaemic index with increased composition of fat and lower proteins compared to gluten-containing meals, resulting in an increasing prevalence of Non-Alcoholic Fatty Liver Disease (NAFLD), weight gain, and alterations of the lipid profile [6].

However, in the 0.3–0.4% of celiac patients, regardless the strict GFD and after excluding other enteropathy causes, the malabsorption and villous atrophy persist, leading to a condition known as refractory celiac disease (RCD) [7]. RCD is divided into two types, RCD-I and RCD-II, based on the quantity of aberrant T cells [8]. In the suspicion of RCD, other causes of increased intraepithelial lymphocytes and villous atrophy (such as *Helicobacter pylori* infection, nonsteroidal anti-inflammatory drugs, and Angiotensin II receptor antagonists) must be excluded [3]. RCD-I is defined with an aberrant T cells percentage lower than 20% [9]. RCD-II is characterized by an aberrant T cell percentage higher than 20% [10]. The latter is considered a pre-lymphoma condition, or low-grade lymphoma because of the high risk of transformation into enteropathy-associated T-cell lymphoma (EATL) [11]. Currently, we have few perspective data on RCD management [8,12,13]. According to the current guidelines, in case of RCD-I, oral budesonide is considered the first-line therapy, while the second-line therapy is represented by immunosuppressants such as thiopurines [14,15,16]. In the literature, only a few cases regarding biological treatment with Infliximab and Small Intestine Release Mesalamine (SIRM) are reported with promising results [17,18]. In RCD-II, chemotherapy with cladribine and autologous haematopoietic stem cell transplantation are considered the first-line therapy [7,19]. In RCD-II, immunosuppressants have a limited role, while oral budesonide seems encouraging [20].

Research into new therapeutic strategies for RCD is necessary since we do not have real therapeutic possibilities. Furthermore, we must consider unintentional gluten ingestion which causes chronic alterations of mucosal integrity and persistent symptoms for which a targeted pharmacological therapy in addition to a GFD could be necessary. 

Currently, a plethora of phase-II trials have been recently published or are ongoing with promising results [21]. Unfortunately, today no novel, effective therapies are available for the treatment of CD and RCD. Thus, outdated drugs often already used for other diseases are our best therapeutic tools in this setting. 

The aim of this review is to analyse and discuss the available adjunctive therapies for the treatment of CD and RCD, as well as to present the novel therapies that are emerging in the current literature for the treatment of RCD and the possibilities to consider using them in future clinical practice.

## 2. Results

At the end of the review process, 2268 articles had been identified and 49 were included in this review (36 studies resulting from the search strategy and 13 from other sources). The PRISMA flow diagram shows the results of the literature search (Supplementary Figure S1). The characteristics of excluded studies are summarized in Supplementary Table S1.

### 2.1. Adjunctive Therapies in Celiac Disease

The standard of care in the management of patients with celiac disease is the GFD; however, clinical, biochemical, and histological recovery are often delayed and could take several months or even years to achieve complete mucosal healing [22]. For this reason, it is a matter of great interest to study therapeutic tools to enhance the rapidity of clinical and histological recovery. In this regard, several conventional drugs including steroids, mesalamine, probiotics, and vitamin D were evaluated (Table 1).

**Figure 1:**
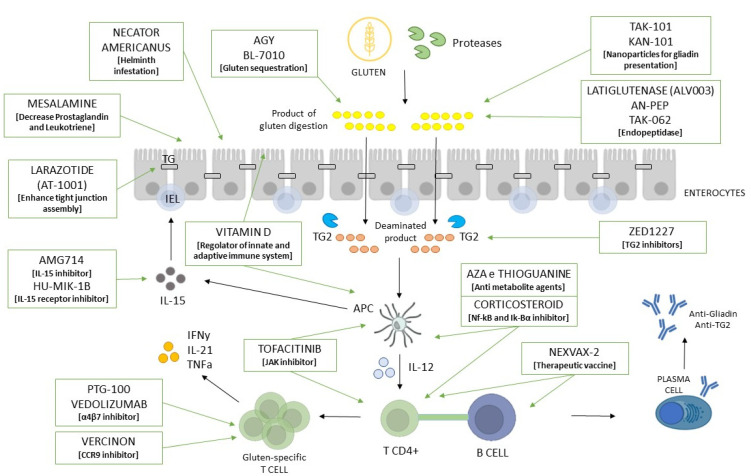
Therapeutic target for novel CD therapies. AZA: Azathioprine; TG2: Transglutaminase II; IL: Interleukin; APC: Antigen Presenting Cells; JAK: Janus Kinase.

Moreover, the advancement of knowledge in the pathogenic processes of CD has helped to identify various targets for future novel therapies and several drug candidates have entered in phase II/III of clinical trials (Figure 1). However, today no one of these novel therapies has proven to be effective.

***Old Adjunctive Therapies in CD***

In this paragraph, we reported an overview concerning therapies with a significant body of evidence concerning CD treatment and not currently involved in RCT. These molecules are approved for the treatment of RCD but not for CD patients because of their suboptimal effectiveness. Moreover, we reported data concerning supplementation therapies used for the management of CD (e.g., Vitamin D).

#### 2.1.1. Corticosteroid

Glucocorticoids are potent inhibitors of T cell activation and cytokine secretion. They mediate their anti-inflammatory responses by binding the intracellular glucocorticoid receptor (GR), also known as the classic GR or GRα, a phosphorylated 92-kDa protein, which is a member of the nuclear receptor superfamily. The GR-mediated transcriptional regulation of specific target genes results in sequence-specific DNA binding which, in turn, inhibits the promoter regions of genes such as Nf-kB and Ik-Bα, which are potent transcription factors for many proinflammatory cytokines and adhesion genes [23].

The very first studies concerning adjunctive therapies in CD patients were all made by trying to evaluate the effect of steroids over the mucosa in untreated celiac patients. In 1970, five patients were treated with prednisolone 10 mg q.d.s. for four to five weeks, obtaining a rapid histological and metabolic response (cell height, lymphoid infiltrate), as much as rapid relapse when the drug was withdrawn [24].

In 1981, Bramble et al., in case-control studies, evaluated two different topical corticosteroids (betamethasone valerate and clobetasone butyrate) in 10 untreated patients while continuing a normal diet for 4 months, showing only minor clinical, biochemical (increased folate, xylose, and faecal fat excretion), brush border enzyme activity (mainly alkaline phosphatase), and histological improvement (enterocyte height and intraepithelial lymphocyte), without justifying their use because of the corticosteroids side effect [25]. Two other more recent studies focused their attention on the effect of another topical steroid, fluticasone propionate, showing a histological and clinical improvement in a small cohort of CD adult patients [26,27]. Mitchinson et al. showed a mean weight gain of 2 kg and a rise in albumin of 5.4 g/L among the 12 adult CD patients included. Moreover, there was a significant improvement in the lactulose/mannitol excretion ratio (*p* less than 0.05) and in all histological variables examined in paired biopsy specimens (surface and crypt intraepithelial lymphocyte/enterocyte and goblet cell/enterocyte ratios and enterocyte height, *p* less than 0.01 or better) [26]. Fluticasone propionate treatment also led to significant increases in the absorptive surface epithelium as shown by an increase in the villus–crypt ratio (*p* less than 0.01), increases in the epithelial cell height (*p* less than 0.01), and two- to three-fold increases in the area and length of the surface epithelium (*p* less than 0.001), as reported by Zaitoun and colleagues [27].

More recently, four single-centre RCTs hypothesized that the addition of a short course of steroids to the GFD in celiac patients might enhance intestinal mucosal recovery and promote faster clinical and histological remission. Two of them, involving 28 and 33 naive celiac patients, respectively, were randomized to either GFD or GFD plus prednisolone (1 mg/kg for 4 weeks) [28,29]. These studies showed no significant clinical differences between the two therapies in terms of clinical symptoms regression (fatigue, weight, height, hemoglobin, number of stools per day, abdominal circumference), IgA anti-tTg seronegativization, and histological regression after 8 weeks and 1 year of treatment, respectively. However, Abbas et al. showed higher histological improvement in the GFD plus Prednisolone group after 6 months. On the other hand, no differences were found after one year of follow-up study [28].

Shaliman et al., in addition, stained duodenal biopsies to assess markers of intrinsic apoptotic pathway (AIF, H2AX, p53), common apoptotic pathway (CC3, M30), apoptotic inhibitors (XIAP, Bcl2), and epithelial proliferation (Ki-67), as well as to compare apoptotic and proliferation indices (PI). After prednisolone therapy, apoptotic markers showed a rapid decline, and there was overexpression of H2AX, CC3, and p53, but also a suppression of mucosal PI, which started rising again after withdrawal of prednisolone. However, the authors underline that prednisolone slows villous regeneration; hence, it should be used only for a short period if applied in patients with celiac disease [29].

Two other RCTs involving 20 and 37 celiac patients, respectively, compared budesonide (6 mg daily for 4 weeks) to GFD alone.

Ciacci et al. showed an increase in body weight, fewer evacuations, and decreased stool weight in CD patients treated with budesonide compared to patients on a GFD alone. An additional analysis investigated the effect of budesonide and gliadin toxic peptides on the intestinal mucosa of celiac patients (an increase of epithelial tyrosine and HLA-DR expression in villi). Treatment with budesonide significantly inhibited the increase of epithelial phosphotyrosine and HLA-DR expression, so it was thus effective in lowering the inflammatory response to gluten [30].

On the other hand, another RCT showed no difference in terms of Marsh grading in CD patients treated with 9 mg/day of budesonide compared to placebo. Moreover, after 8 and 52 weeks there were no histological differences between the two strategies (histological remission at 52 weeks: 42% budesonide vs. 33% GFD; *p* = 0.74) [31].

In conclusion, the high-quality RCTs and the overall body of evidence showed little to no effect of the steroids compared to GFD on the improved histological outcome (such as an increase in the villus with the crypt and the epithelial cell height) or clinical symptoms regression (such as fatigue, weight, and height). Moreover, these effects are limited in time and burdened by relevant side effects. Thus, today the use of steroids is not approved for the management of celiac disease. 

#### 2.1.2. Mesalamine

Mesalamine is the active moiety of sulfasalazine, which is metabolized to sulfapyridine and mesalazine. The exact mechanism of mesalamine is unknown, but it is speculated that mesalamine decreases the synthesis of prostaglandin and leukotriene, thus modulating the associated inflammation [32].

The effects of mesalamine in an organotypic culture from biopsies of newly diagnosed CD patients were evaluated in an in-vitro study. Three cohorts (naive-CD, GFD-CD patients, and a control group) were included. The samples treated with mesalamine for 24 hours showed a decrease in the typical CD inflammatory response rather than GFD, and there was a significant decrease in the SODs/Catalase ratio (*p* < 0.005), the NF-kB protein levels (*p* < 0.005), its target gene NOS2, and lipid peroxidation (*p* < 0.005). Furthermore, mesalamine is able to upgrade PPARγ expression as with the control samples [33]. However, the lack of human studies and in particular RCTs does not justify mesalamine use in CD patients. 

#### 2.1.3. Vitamin D

Vitamin D is a group of fat-soluble steroids responsible for increasing intestinal absorption of calcium, magnesium, and phosphate, and for many other biological effects involving the health of bones, digestive tract, and other organs [34]. Growing evidence on the effect of Vitamin D in immune modulation and its implications in immune-mediated diseases, and in particular in inflammatory bowel disease (IBD), are emerging [35].

Among micronutrients, Vitamin D could have a role in CD due to its importance in the regulation of both innate and adaptive immune system activity.

A single study concerning Vitamin D supplementation on enteropathic mice after the electronic search was included. The histopathological evaluation of the intestinal mucosa showed that mice ongoing cholecalciferol treatment had low grade or normal histological features compared to celiac mice; furthermore, a dose-dependent increase in villi length was highlighted in mice receiving the cholecalciferol. Thus, cholecalciferol was able to significantly reduce the presence of intestinal mucosal lesions and increase villi length (with a dose-dependent effect) [36].

The lack of human studies does not justify the use of Vitamin D in CD patients as adjunctive therapy outside the case of bone metabolism indication. Thus, stronger evidence deriving from RCTs involving human CD patients is needed.

***New Adjunctive Therapies in CD***

In this paragraph, we report studies concerning new hypothetical therapies involved in ongoing or recently published RCTs. However, today any of the following therapies is recommended in the current guidelines.

#### 2.1.4. Tight Junction Modulation

Currently, the only drug in phase III is larazotide acetate (also known as AT-1001 and INN-202), which is a synthetic, eight-amino acid peptide that decreases intestinal permeability, enhancing tight junction assembly and actin rearrangement in vivo and in vitro [37].

In a double-blind, randomized, placebo-controlled trial, following acute gluten exposure, the AT-1001 group showed a lower increase in intestinal permeability compared to the placebo. Gastrointestinal symptoms were less frequently detected in the AT-1001 group compared to the placebo group (*p* = 0.018) [38]. 

A phase II randomized trial assessed the efficacy and tolerability of larazotide acetate during gluten challenge. Larazotide acetate reduced gluten-induced immune reactivity and symptoms; however, no significant difference in the lactulose-to-mannitol (LAMA) ratio between larazotide acetate and placebo was observed [39]. In the last published randomized trial, larazotide acetate reduced symptoms in CD patients on a GFD better than a GFD alone [40]. The primary endpoint was the reduction of symptoms in the larazotide group with the 0.5-mg dose of larazotide acetate, compared to placebo (*p* = 0.005). The 0.5-mg dose showed an effect on exploratory endpoints including a 26% decrease in celiac disease patient-reported outcome symptomatic days (*p* = 0.017), a 31% increase in improved symptom days (*p* = 0.034), a 50% or more reduction from baseline of the weekly average abdominal pain score for 6 or more of 12 weeks of treatment (*p* = 0.022), and a decrease in the non-gastrointestinal symptoms of headache and tiredness (*p* = 0.010). However, in 2022 a phase 3 clinical trial was suspended after an interim analysis showed no significant effectiveness [21]. More high-quality data are needed to consider this molecule for possible adjunctive therapies in CD.

#### 2.1.5. Transglutaminase II Inhibitors

Transglutaminase II (TG2) inhibition blocks gliadin-induced proliferation of gliadin-specific T cells and prevents the increase of activated T cells in patient small-bowel mucosal biopsy sample organ culture [41]. Moreover, the inhibition of TG2 modulates intestinal epithelial permeability functions in vitro [42].

An elegant phase-II clinical trial involving 160 CD patients in remission assessed the effectiveness of the TG2 inhibitor (ZED-1227), orally administered (at a daily dose of 10, 50, or 100 mg) after six weeks of gluten challenge. Treatment with ZED1227 at all three dose levels attenuated gluten-induced duodenal mucosal injury compared to placebo (*p* < 0.001) with a similar rate of mild adverse events among all groups [43].

These data make the TG2 inhibitor into promising molecules to be subjected to further studies.

#### 2.1.6. Endopeptidases

Oral enzymatic therapy is a widely investigated therapeutic approach. It is based on the inactivation of immunogenic gluten peptides in the human gastrointestinal tract by peptidase supplementation, minimizing the quantity of gluten peptides that reach the small intestine. The administration of exogenous endopeptidases could be a valuable novel strategy to prevent the immunogenicity of gluten. Several RCT and observational studies have evaluated this potential mechanism of action. The most promising molecule is latiglutenase.

ALV003, or latiglutenase, which belongs to the prolyl endopeptidase family, is the most extensively investigated enzyme mixture in human trials. ALV003 is an orally administered mixture of two gluten-degrading proteases which are activated in the acidic environment of the stomach [44].

A phase I study evaluated the efficacy of TAK-062 under simulated gastric conditions in vitro and in healthy participants, showing that it is well tolerated and effectively degrades large amounts of gluten [45].

However, a phase II trials did not show differences between latiglutenase and placebo in reducing villous atrophy or improving symptoms in CD patients [46].

A post hoc analysis of a trial involving symptomatic CD patients on a GFD (for at least one year before randomization) analysed the effectiveness of 12 weeks of oral latiglutenase treatment. The authors showed a reduction of symptoms in the subgroups of seropositivity, but not in seronegative, CD patients considering Celiac Disease Symptom Diary (CDSD) and the Patient Global Impression-Symptoms (PGI-S) scores [47,48].

Another phase II randomized clinical trial showed symptomatic improvement using latiglutenase, also reducing gluten-induced intestinal mucosal damage [49]. The efficacy of aspergillus niger prolyl endoprotease (AN-PEP) was studied in a randomized pilot study, but the improvement in histological evaluation according to Marsh classification (the primary endpoint) was not met because no significant change in the degree of mucosal damage was observed [50].

Generally, the overall body of evidence concerning endopeptidases did not reveal significant effectiveness of these therapies as adjunctive treatment in CD patients.

#### 2.1.7. Gluten Sequestration

Another novel therapeutic approach involves sequestering gluten in the intestinal lumen before its digestion into immunogenic peptides. This approach includes gliadin-targeting antibodies and polymeric binders (AGY and BL-7010). The efficacy of oral egg yolk anti-gliadin antibody (AGY) was studied in a 6-week, open-label, single-arm study.

In this study, to assess quality of life of the patients the OptumTM Short-Form Quality-of-life Questionnaire Version 2 (SF-36v2) was used; it is a common validated tool to evaluate patient-reported health including general health (GH), physical functioning (PF), role physical (RP), bodily pain (BP), vitality (VT), social functioning (SF), role emotional (RE), and mental health (MH), as well as two composite elements, physical component summary (PCS) and mental component summary (MCS) [51].

At the end of the follow-up study, the included patients presented fewer celiac-related symptoms and improved their quality of life when taking AGY compared to the run-in period [52].

An ongoing randomized, double-blind, placebo-controlled, crossover trial still at the stage of recruiting aims to evaluate the safety and efficacy of AGY in CD patients. This data could help to clarify the efficacy of AGY as adjunctive therapy in CD patients [NCT 03707730].

Thus, more high-quality data are needed to consider this molecule for possible therapeutic options in CD patients.

#### 2.1.8. Nanoparticles for Gliadin Presentation

In order to modulate the immuno-tolerance in CD caused by the dysregulation of T regulatory cells, the nanoparticles for gliadin presentation have been studied. Negatively charged poly(dl-lactide-co-glycolide) (PLGA)-antigen (Ag) nanoparticles have been developed to deliver specific antigens that induce tolerogenic inhibition via a non-inflammatory process [53]. In a phase II RCT, 33 CD patients completed the 14-day gluten challenge. TAK-101 induced an 88% reduction in change from baseline in interferon-γ spot-forming units vs. placebo (2.01 vs. 17.58, *p* = 0.006). Moreover, TAK-101 reduced changes in circulating α4β7+CD4+ (0.26 vs. 1.05, *p* = 0.032) [53].

These data make the nanoparticles for gliadin presentation into promising molecules to be subjected to further studies.

#### 2.1.9. IL-15 Signalling

IL-15 and its downstream signalling route are interesting targets as IL-15 is upregulated in both intestinal epithelial cells and lamina propria in active CD [54]. Results have been reported for AMG 714, a fully human monoclonal antibody that binds all forms of IL-15 inhibiting their functions [55]. Therefore, tofacitinib, a JAK2/3 inhibitor, has demonstrated some beneficial effects in transgenic mice overexpressing human IL-15, which mimics RCD [56].

However, today outside the few available data concerning RCD patients (such as the ongoing trial, Eudra CT: 2018-001678-10), these therapies have not found a body of evidence to support their use in CD.

#### 2.1.10. Therapeutic Vaccine

Another explored therapeutic possibility was represented by Nexvax2, a therapeutic vaccine composed of three gluten peptides encompassing five HLA-DQ2-restricted epitopes frequently recognized by gluten-specific T-cells [57]. However, its lack of effectiveness has already been proven [21].

Phase 1 studies have shown the safety of the vaccine after intradermal administration, even if it induced diarrhoea and nausea as side effects [58].

Another phase 1 RCT with increasing doses (from 60 µg to 150 µg twice weekly for 8 weeks) did not show an improvement in small bowel histology in 108 CD patients exposed to gluten [59].

Finally, a phase 2 study aimed to document the bioavailability of Nevax2 peptides after subcutaneous and intradermal dosing, as well as the tolerability and ability of subcutaneous dosing to induce non-responsiveness to Nexvax2 peptides, and it showed that subcutaneous and intradermal dosing of Nexvax2 yield similar bioavailability of constituent peptides; therefore, subcutaneous dose escalation avoids an immune response to dominant gluten epitopes [60].

Although this vaccine could represent an interesting novelty, it has not been supported by the clinical data in the current literature, and indeed it is not included in guidelines for RCD treatment.

#### 2.1.11. Prebiotics and Probiotics

CD is associated with intestinal microbiota alterations. The administration of probiotics and prebiotics could be a promising method of restoring gut homeostasis in CD. The hypothetical mechanism of action of *Bifidobacterium longum* CECT 7347 was investigated in a study including paediatric patients on GFD with newly diagnosed CD. Anthropometric parameters, lymphocyte phenotyping, cytokines (TNF-a, interferon-γ, IL-13, and IL-10), gut microbiota composition, and serum IgA quantification were assessed at the baseline and after three months. The results showed that oral administration of *Bifidobacterium longum* significantly reduced both the CD3 T lymphocyte population and the faecal IgA concentration. There was also a decrease in the levels of TNF-a, although this was not statistically significant [61].

A placebo-control, double-blind, randomized study including adult patients with positive serology for CD showed a reduction in symptoms, particularly constipation, in patients treated with *Bifidobacterium infantis* [62]. Furthermore, in children *Bifidobacterium infantis* decreases Paneth cell counts and the expression of α-defensin-5 in CD, both of which are also related to the modulation of innate immunity [63].

Francavilla et al., in a prospective, double-blind placebo-control study, showed that the administration of a probiotic mixture (five strains of lactic acid bacteria and bifidobacteria) determined an improvement in CD symptoms measured via questionnaires such as the IBS-SSS (Irritable Bowel Syndrome Severity Scoring System) and the GSRS (Gastrointestinal Symptom Rating Scale) [64].

Interestingly, the administration of oligofructose-enriched inulin (that significantly increases bifidobacterium count) resulted in an improvement in the consistency of stool in children on a GFD compared to placebo suggesting a potential role of the prebiotic [65].

A recent RCT has evaluated the effectiveness of probiotics in a population of CD paediatric patients. Prolonged oligofructose-enriched inulin (Synergy 1) was administered and the characteristics and metabolism of intestinal microbiota in CD children on GFD compared to placebo were evaluated. The quantitative gut microbiota characteristics and short-chain fatty acids (SCFAs) concentration were analysed showing a moderate effect on the qualitative characteristics of faecal microbiota [65].

Although encouraging, the data concerning treatment with probiotics shows the evidence is significantly heterogeneous in terms of outcomes measured (e.g., characteristics of microbiota, symptoms using different or no validate score, cytokines). Thus, RCTs of higher methodological quality are needed, especially regarding the outcomes measured.

### 2.2. Management of Slow Responder CD Patients

Between 7% and 30% of adult CD patients are slow responders because they have persistent symptoms, signs, or laboratory abnormalities of CD despite at least 6–12 months of GFD [66,67].

These patients are classified as slow-responders and not as non-responders because most of them will improve afterward on GFD or they will show a treatable cause for the absence of a response.

Despite a large number of non-randomized studies and RCTs, none of the above-mentioned therapies are approved in the current CD guidelines. Thus, the correct management of these patients’ needs requires first of reconsidering the diagnosis of CD by reviewing the small-bowel histology and serology obtained at the time of diagnosis, because alternative diseases and treatments must be considered in this case [67,68].

In patients with confirmed CD, the inadvertent ingestion of gluten is the most common cause of slow response (35–50%). In these cases, celiac serology is helpful if positive when the cause of slow response is gluten exposure. However, a normal serology does not exclude intermittent or low-level gluten ingestion [8,67]. Obviously, the accidental ingestion of gluten is often hard to assess. For this purpose, the detection of gluten peptides in urine and faeces has been developed, so it could be considered a novel diagnostic tool [69,70,71].

Therefore, another evaluation should be to look for other food intolerances (e.g., lactose or fructose) and medications [72].

Once dietary causes have been excluded, duodenal biopsies should be repeated. A Marsh 0–1 small bowel histology suggests other aetiologies (IBS, microscopic colitis, food intolerances, small intestinal bacterial overgrowth, or exocrine pancreatic insufficiency) [8,73].

CD-like enteropathy has also been reported in association with olmesartan, losartan, and mycophenolate [72]. Thus, beyond the anecdotal report shown in this review, no adjunctive pharmacological therapy is currently indicated for CD treatment.

### 2.3. Adjunctive Therapies in Refractory Celiac Disease

RCD, although rare, represents a hard challenge for clinical management. It is defined as the persistence of symptoms and signs of malabsorption and villi atrophy, despite adequate compliance with GFD for more than 12 months, in the absence of other causes of non-responsive treated CD and overt malignancy (like overt lymphoma) [14].

RCD diagnosis is most frequent in women after the fifth decade and it is characterized by persistent diarrhoea, abdominal pain, involuntary weight loss, multiple vitamin deficiencies, anaemia, and fatigue [8,14].

There are two types of RCD: type I, defined as the presence of <20% monoclonal aberrant T lymphocytes; type II, defined by >20% monoclonal T cells. The RCD-II is also defined as pre-lymphoma (Pre-EATL) or low-grade lymphoma. It is characterized by a high risk of transformation in enteropathy-associated T cell lymphoma (EATL) [14].

The current guidelines suggest oral budesonide for RCD-I only. Although recently, few encouraging data concerning RCD-II are available. Steroids, mesalamine, immunomodulators, biologicals, and small molecules have been used in the treatment of RCD (Table 2).

***Old Adjunctive Therapies in RCD***

In this paragraph, we reported approved therapies and drugs with a more solid body of evidence concerning the treatment of RCD.

**Table 1:** Main findings of included studies concerning the available adjunctive treatment of celiac disease (non-randomized studies of intervention and RCT).

Studies	Study Design	Population	Follow-Up	Biochemical Outcome		Histological Outcome		Clinical Outcome	
				Analysed Treatment: Prednisolone					
Alfred J Wall, 1970 [24]	Prospective	5 CD	5 weeks	Faecal fat excretion	n.a.	Enterocyte height, intraepithelial lymphocytes	n.a.	n.a.	n.a.
Shalimar 2012 [29]	RCT	33 CD (16 GFD and prednisolone vs. GFD)	2 months	h2ax	*p* = 0.04	n.a.	n.a.	n.a.	n.a.
				P53	*p* = 0.15				
				AIF	*p* = 0.7				
				M30	*p* = 0.9				
				CC3	*p* = 0.6				
				KI-67	*p* = 0.5				
Abbas, 2018 [28]	RCT	28 CD (14 GFD and prednisolone vs. GFD)	12 months	n.a.	n.a.	Marsh	*p* = 0.08	Number stools Weight	*p =* 0.22 *p =* 0.9
			**Analysed treatment: Betamethasone valerate and clobetasone butyrate**						
Bramble, 1981 [25]	Prospective	10 CD	12 months	Xylose excretion Faecal fat excretion	*p* < 0.01 *p* < 0.02	Intraepithelial lymphocytes enterocyte height	*p* < 0.01 *p* < 0.01	n.a.	n.a.
				**Analysed treatment: Fluticasone propionate**					
Mitchison, 1991 [26]	Prospective	12 CD	6 weeks	Albumin, Hb Lactulose/mannitol excretion ratio	*p* < 0.01 *p* < 0.05	Intraepithelial lymphocytes Enterocyte height Enterocytes/GR ratio Alkaline phoshatase, lactase, sucrase activities	*p* = 0.002 *p* < 0.001 *p* = 0.002 *p* < 0.05	Weight gain, bowel frequency, stool consistency	*p* < 0.05
Zaitoun, 2007 [27]	Prospective	10 CD	6 weeks	n.a.	n.a.	Reduction intraepithelial lymphocyte Epithelium surface area	*p* < 0.01 *p* < 0.001	n.a.	n.a.
				**Analysed treatment: Bifidobacterium longum CECT 7347**					
Olivares, 2014 [61]	RCT	33 CD (17 B. Longum vs. 16 placebo)	3 months	Decreased peripheral T CD3+ Content of sIgA in stools	*p* = 0.004 *p* = 0.011	no data	n.a.	Height percentile increases	*p* = 0.048
				**Analysed treatment: Mesalamine**					
Benedetti, 2018 [33]	In vitro study	20 organotypic culture	Incubated for 24 h	n.a.	n.a.	Decrease in SOD/catalase ratio 4HNE Decrease NFKb and NOS2 in 5ASA-CD cultures	*p* < 0.0005 *p* < 0.005 *p* < 0.005	n.a.	n.a.
				**Analysed treatment: cholecalciferol (vitamin D3)**					
Trasciatti, 2022 [36]	Prospective (murine model)	103 CD (90 cholecalciferol; 13 placebo)	12 weeks	n.a.	n.a.	Villi lengthCD3 ZO-1 8	*p* < 0.0001 *p* = 0.002 (villus) *p* = 0.027 (crypt)	n.a.	n.a.
			**Analysed treatment: Budesonide**						
Ciacci, 2009 [30]	RCT	20 CD (10 GFD and Budesonide vs. GFD)	1 months	HLA-DR Thirosine phosphorylase ICAM-1 COX-2	*p* < 0.005 no data no data *p* < 0.05	n.a.	n.a.	Stools weight	0.016
Newnham, 2021 [31]	RCT	37 CD (19 Budesonide vs. Placebo)	12 months	n.a.	n.a.	Marsh	*p* = 0.032	n.a.	n.a.

mean; n.a.: not available; GFD: gluten free diet; CD: celiac disease; RCT: randomized controlled trial.

#### 2.3.1. Corticosteroid

The current American Gastroenterological Association Guidelines (AGA) recommend corticosteroids as first-line therapy in either type 1 or type 2 refractory celiac disease. Open-capsule budesonide or, if unavailable, prednisone, are the medications of choice and should be used in RCD treatment [74].

Several studies have evaluated the efficacy of treatment with budesonide in patients affected by RCD, reporting a clinical improvement. [75]. On the other hand, few studies reported an improvement of intestinal villous atrophy [76].

A retrospective study including 13 and 43 patients with RCD-I and RCD-II, respectively, showed a clinical and histological response following treatment with oral budesonide at a dosage of 9 mg/day. The clinical and histological response was observed both in patients with RCD-I and those with RCD-II, regardless of previous treatment with immunosuppressants (azathioprine) or systemic steroids [20].

Similar observations come from an open-labelled non-controlled study [77]. Among 29 RCD patients, a complete clinical response was observed in approximately 80% of the patients treated with budesonide alone. However, there was no improvement in the duodenal biopsy over the study period and no significant side effects were reported. A case series showed a clinical response in seven out of nine RCD-included patients wherein six of these had been switched after induction with 20–40 mg/day of prednisone for four months to therapy with budesonide 9 mg/day, while one of them had immediately started treatment with budesonide at the same dosage. However, duodenal histology improvement was observed only in three patients [78].

#### 2.3.2. Mesalamine

The effectiveness of mesalamine in RCD was shown in an open-label therapeutic trial that was published in 2011. Four patients treated with small intestinal release mesalamine (SIRM) and six patients treated with SIRM and oral budesonide were included. An improvement in global symptoms and a decrease in the daily number of bowel movements were shown in 60% of the included patients (50% complete response and 10% partial response, respectively). Considering the small sample size, no difference was found between the two groups [79].

#### 2.3.3. Immunomodulator

The immunomodulators studied in RCD are Azathioprine, Thioguanine, and steroids.

Azathioprine is a prodrug of 6-mercaptopurine, a thiopurine, considered an immunosuppressant that is used to treat autoimmune and inflammatory conditions.

Tioguanine is a 2-aminopurine that incorporates into DNA and inhibits its synthesis, so it has a role as an antineoplastic and antimetabolite agent.

The first experience on immunomodulators in RCD was published in 1976. In a case report, a CD woman who did not respond to a GFD was successfully treated with a prednisone-azathioprine combination. The patient was initially treated with prednisone 40 mg/daily. However, after steroid de-escalation, serious clinical relapse occurred. Thus, after the introduction of azathioprine (2 mg/kg/day), it was possible to reduce the dose of steroids, obtaining clinical remission with relative histological improvement [80].

One open-label trial conducted by Goerres et al. evaluated the therapeutic effects of azathioprine in 18 patients with RCD [81]. All patients were treated with oral azathioprine for 52 weeks and with prednisone used as induction therapy for 6 weeks and then tapered. Eight type RCD-I patients completed the treatment, whereby 8/10 (80%) of them achieved a reduction of intraepithelial lymphocytes and four achieved normal villous architecture after treatment. Among type RCD-II, only five of them completed the treatment. One showed an improvement in the histological picture with the persistence of high lymphocytic infiltration, while the others did not improve with the treatment and subsequently died due to the onset of EATL. In conclusion, this study suggests the combination of azathioprine-prednisone as a valid therapeutic option in patients with RCD-I, while in patients affected by RDC-II it is observed that the treatment is useless and may rather compromise the performance status of the patients [81].

A retrospective study, including ten patients affected by RCD-I treated with tioguanine, showed a complete histological response in seven patients (78%). Two patients did not show a histological response after 12–14 months of follow-up study. Eight patients were treated with tioguanine as first-line treatment, whereas four patients received tioguanine as second-line treatment due to intolerance or resistance to azathioprine or corticosteroid dependency. Among the included patients, one died after 4 months of therapy due to the progression of RDC-I with severe diarrhoea and severe metabolic dysregulation. Muscle spasms were present in one patient (who withdrew tioguanine therapy) [82].

***New Adjunctive Therapies in RCD***

Considering the low incidence of RCD, RCTs are not available and are very difficult to set. Thus, only a few anectodical experiences exist in the literature.

#### 2.3.4. Biological Therapy and Small Molecules

Biological therapy studied in RCD is represented by Infliximab, a monoclonal antibody composed of human constant and murine variable regions that binds specifically to human tumour necrosis factor alpha (TNFα).

The small molecule is represented by Tofacitinib, an inhibitor of Janus kinases, a group of intracellular enzymes involved in signalling pathways that affect haematopoiesis and immune cell functions.

Three case reports have investigated the efficacy of anti-TNF-α (Infliximab) alone or in combination with other drugs in RCD treatment. 

In the first study, published in 2002, after an unsatisfactory response to azathioprine and prednisolone, remission was achieved with an infusion of Infliximab (5 mg/kg over 2 hours) and maintained using azathioprine (2 mg/kg) [83].

Similar results were reported in a case report published in 2005 [84]. Clinical, laboratoristic, and histological responses were achieved after treatment with Infliximab (5 mg/kg) and prednisolone (30 mg daily). Prednisolone and azathioprine (100 mg daily) were used for the maintenance treatment. On the other hand, another study reported that treatment with 5 mg/kg of Infliximab improved malabsorptive symptoms and signs without obtaining healing of mucosal lesions [85].

In a pilot, non-randomized, open-label study, 10 RCD patients were treated with a recombinant human IL-10 (rHu-IL-10) at a dosage of 8 mcg/kg for 3 months. Marsh classification was the main outcome evaluated. Two out of ten patients dropped out due to severe side effects. At the end of the follow-up study, the histology was unchanged in five patients and improved in two patients. Only three out of eight patients reported a clinical response. Interestingly, after IL-10 withdrawal, the symptoms recurred in all responsive patients [86].

In a 2022 case report, a patient diagnosed with RCD-II/ulcerative jejunitis (previously treated unsuccessfully with budesonide, Infliximab, and cladribine) was treated with Tofacitinib (10 mg twice a day, then lowered to 5 mg twice a day). Marsh classification improved from III to I-II and laboratory parameters and clinical symptoms also improved [87].

### 2.4. Management of Refractory Celiac Disease Patients

Treatment for RCD is nowadays based on nutritional support and pharmacological intervention.

As demonstrated in studies included in our review, the most effective medical treatment in RCD I patients are steroids. The first therapeutic steps consist of open capsule/non-slow-release budesonide, 3 mg, 3 times a day, for at least 3 months [20,77].

In case of response, Azathioprine (2–2.5 mg/kg/day) or 6-Thioguanine can be administered as maintenance therapy, and after 3 months duodenal biopsies may be repeated. After that, an annual biopsy with aberrant intra-epithelial lymphocyte evaluation represents the best follow-up regimen. After 2–3 years of complete response, Azathioprine could be suspended [82].

In case of failure to respond, RCD I misdiagnosis must be excluded and thiopurine dosage may be optimized.

In our review, only three studies have evaluated mesalamine, infliximab, or azathioprine-prednisone combination as a viable therapy for RCD I so these drugs may be considered as therapeutic alternatives although the evidence is very uncertain [17,79,81].

Five days of purine analogue inhibitors, such as cladribine (2-CDA) or fludarabine, at a dosage of 0.15 mg/kg/day to be repeated in case of relapse after 6–12 months, can also be considered, as shown in other studies included in this paper [19,88]. If symptoms persist after 2-CDA therapy, RCD II misdiagnosis must be ruled out and, if RCD II is confirmed, a high dose of 2-CDA therapy followed by auto-stem cell transplantation may be initiated [89]. According to the topical literature, the reduction of aberrant T cells is not showed in patients treated with auto-stem cell transplantation, and long-term outcomes, notably the onset of EATL, are still awaited [90].

Currently, auto-stem cell transplantation is indicated only in symptomatic patients and not in asymptomatic patients trying to eliminate aberrant intra-epithelial lymphocytes [14].

Tofacitinib (Jak-3 inhibitor) and anti-IL15 monoclonal antibodies are still considered experimental treatments since they have been clinically tested only in Phase 2 and Phase 3 studies [91,92].

**Table 2:** Main findings of included studies concerning the available adjunctive treatment of refractory celiac disease (non-randomized studies of intervention and RCT).

Studies	Study Design	Population	Follow-Up	Biochemical Outcome		Histological Outcome		Clinical Outcome	
				Analysed Treatment: Steroid					
Mukewar, 2017 [20]	Retrospective	52 RCD	17 Months	n.a.	n.a.	Marsh classification	Histological improvement in 60% of patients with RCD-I; 55% in RCD-II *p* = 0.19	Stool frequency, Weight gain	Clinical improvement in 68% of patients with RCD-I77% in RCD II *p* = 0.48
Brar, 2007 [77]	Retrospective	29 RCD	7 Months	n.a.	n.a.	n.a.	n.a.	Stool frequency	Clinical improvement
				**Analysed treatment: Steroid-Azathioprine**					
Goerres, 2003 [81]	Prospective	10 RCD-I; 8 RCD-II	12 Months	Hemoglobin, serum albumin; folic acid	Biochemical improvement	Marsh classification	Histological improvement in RCD-I	Abdominal pain, diarrhoea and weight loss, prednisone (mg/day)	Clinical improvement
Tack, 2012 [82]	Prospective	12 RCD-I	24 Months	Hemoglobin concentration, serum albumin,	Biochemical improvement	Marsh classification	Histological improvement	Median weight, BMI, prednisone (mg/day)	Clinical improvement
				**Analysed treatment: SIRM-Budesonide**					
Jamma, 2011 [79]	Retrospective	10 CD (4 SIRM vs. SIRM and Budesonide)	67.2/62.5 * weeks	n.a.	n.a.	n.a.	n.a.	Global symptoms; bowel movements/ day	Clinical improvement
				**Analysed treatment: rHu-IL-10**					
Mulder, 2001 [86]	Prospective	10 CD	9 Months	n.a.	n.a.	Marsh classification	Very limited efficacy	n.a.	n.a.

* mean; n.a.: not available; SIRM: small intestinal release mesalamine; RCD: refractory celiac disease; CD: celiac disease.

## 3. Materials and Methods

Four authors independently reviewed the literature (ES, SF, MM, CR). Abstracts and full texts were screened in order to include eligible articles. An electronic search was performed using PubMed (Medline) and Scopus. This research included the combination of the following Medical Subject headings (MeSH) and keywords:

(“celiac disease”[MeSH Terms] OR “refractory celiac disease”[All Fields] OR “celiac disease”[All Fields]) AND (“molecular therapies”[All Fields] OR “molecular therapy”[All Fields] OR (“mesalamine”[MeSH Terms] OR “mesalamine”[All Fields] OR “steroids”[MeSH Terms] OR “steroids”[All Fields] OR “steroid”[All Fields]) OR (“adrenal cortex hormones”[MeSH Terms] OR (“Zonulin”[All Fields]) OR “Transglutaminase”[All Fields] OR “Endopeptidases”[All Fields])).

All randomized clinical trials (RCTs) and non-randomized studies of intervention (NRSI) that evaluated histological, clinical, or biochemical outcomes were considered eligible. In case of a lack of both RCT and NRSI, case reports were considered. Studies including both adults and paediatric patients were considered as includible in this review. Each of the relevant publications (previous review articles and included studies), reference sections, and other relevant studies from other sources were also screened for other relevant publications. Pertinent abstracts from the United European Gastroenterology Week conference (UEGW) and Google Scholar were also screened.

## 4. Conclusions

In recent years, the increasing incidence of CD has stimulated the search for new therapeutic strategies in order to improve the quality of life of CD patients. The need for new therapies aims on the one hand to avoid the limitations of the social relationships of GFD and on the other hand the lack of therapies for RCD. Today, according to current guidelines, the GFD remains the only effective treatment for the CD. Steroids, mesalamine, immunomodulators, and more recently the biological therapies have been used in the treatment of RCD, but with unexciting results [3,14]. Therefore, research into new therapeutic strategies for RCD is necessary since we do not have real therapeutic options for these patients.

Concerning the CD, the numerous recently published phase II-III studies of new drugs analysed in this review show encouraging results, although evidence of their clinical efficacy is still limited. Moreover, despite encouraging data concerning corticosteroids, the heterogeneity in the reported outcomes and the balancing between the effective and side effect make these therapies unattractive.

Thus, according to the current guidelines, no drug therapy is better than GFD in CD, and the current evidence does not support any adjunctive therapy. In slow responders, despite the large number of studies about old and new therapies, no drugs are approved in the current guidelines. Few encouraging data concerning treatment with probiotics have emerged. However, the evidence is significantly heterogeneous in terms of outcome measured, and the quality of evidence is suboptimal. Few novel therapies, among the plethora of newly reported drugs, may be revealed as useful adjunctive therapeutic strategies in CD patients such as Transglutaminase II inhibitors, gliadin-targeting antibodies, and polymeric binders. However, high methodological RCTs and clinical data are needed.

Finally, there is also no curative treatment for RCD, and management consists of a combination of nutritional support and immunosuppressive drugs which are supported by few clinical data. The current AGA guidelines suggest corticosteroids as first-line therapy in either type 1 or type 2 refractory celiac disease (ideally the open-capsule budesonide). Patients with refractory celiac disease without response to steroids may benefit from referral to a centre with expertise for management or evaluation for inclusion in clinical trials [74].

Despite some encouraging data, this review suggests that in this topic further studies with larger populations and better clinical evidence are needed.

## Data Availability

Not applicable.

## Supplementary Materials

The supporting information can be downloaded at: https://www.mdpi.com/article/10.3390/ijms241612800/s1.

## Abbreviations

CD	Celiac Disease
RCD-I/II	refractory celiac disease type I/type II
GFD	Gluten-Free Diet
NAFLD	Non-Alcoholic Fatty Liver Disease
EATL	Enteropathy-Associated T-cell Lymphoma
RCT	Randomized Clinical Trial
NRSI	Non-randomized study of intervention
SIRM	Small Intestine Release Mesalamine
TG2	Tranglutaminase II
