# Supplementary material for: Old and New Adjunctive Therapies in Celiac Disease and Refractory Celiac Disease: A Review
Source: Int J Mol Sci. 2023 Aug 15;24(16):12800. doi: 10.3390/ijms241612800 (PMC10454405; doi:10.3390/ijms241612800)
Supplement: Supplementary file 1 [file ijms-24-12800-s001.zip › ijms-2444526-supplementary.pdf]

SUPPLEMENTARY MATERIALS

Supplementary Figure S1: PRISMA flow diagram

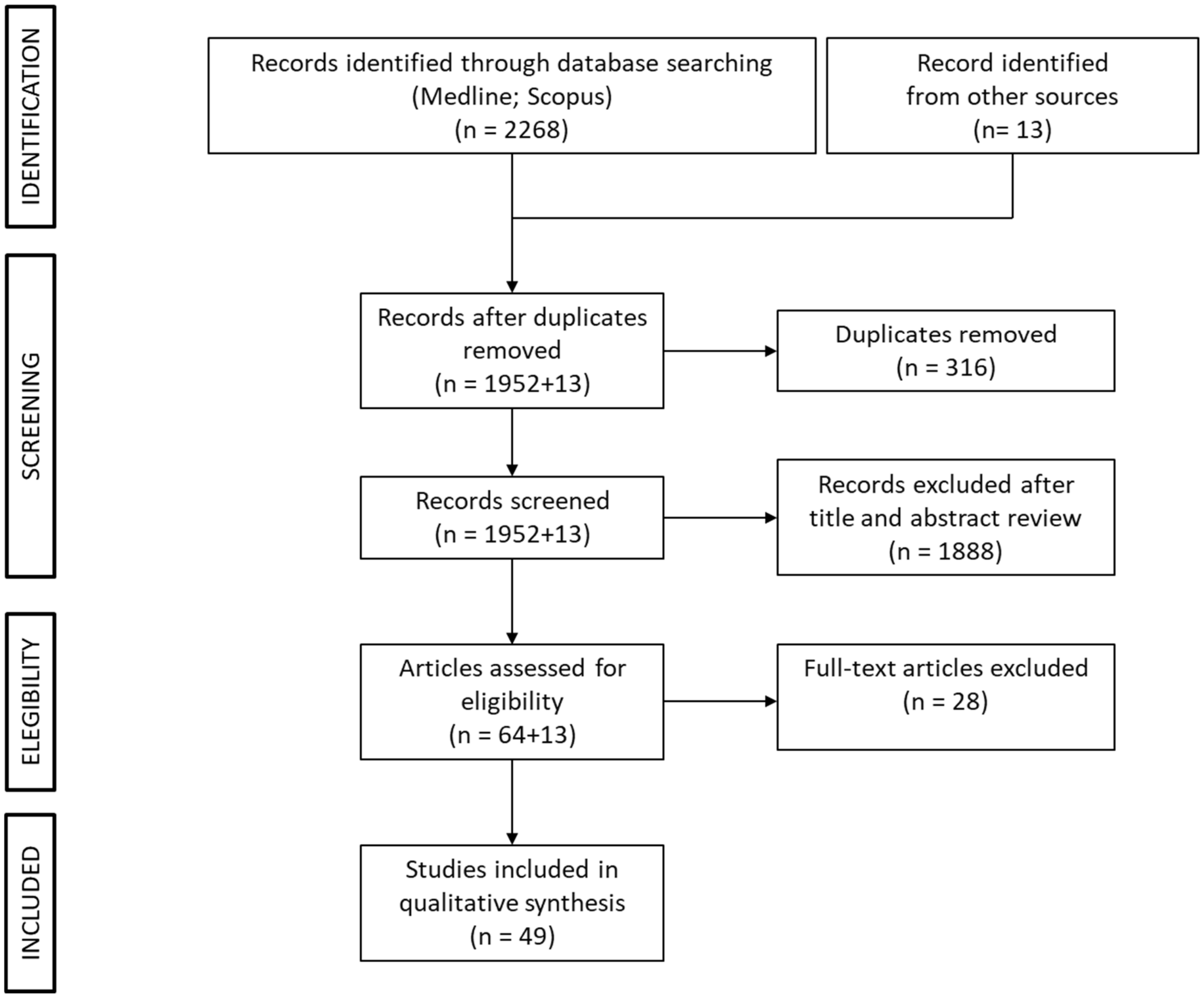

Supplementary Table S1: Excluded studies

	**Reference**	**Reason of Exclusion**
**1**	**Hartl C. 2021**	Outcome of interest not assessed
**2**	**Yoosuf S. 2019**	Review article
**3**	**Lopetuso LR. 2015**	CD patients not included
**4**	**Hasan A. 2013**	Outcome of interest not assessed
**5**	**Sitkin SI. 2013**	Outcome of interest not assessed
**6**	**Latorre M.**	Review article
**7**	**Malamut G. 2010**	Outcome of interest not assessed; not CD therapies
**8**	**Green PHR. 2009**	Outcome of interest not assessed; not CD therapies
**9**	**Stuart BM. 1998**	Outcome of interest not assessed
**10**	**Kumar PJ. 1973**	Outcome of interest not assessed
**11**	**Tukaj S. 2017**	Outcome of interest not assessed
**12**	**Therrien A. 2020**	Outcome of interest not assessed
**13**	**Radovic NP. 2011**	Outcome of interest not assessed
**14**	**Kunduhoglu B. 2021**	Outcome of interest not assessed
**15**	**Marietta E.**	CD patients not included
**16**	**Shah S. 2022**	Outcome of interest not assessed

**17**	**Weber M. 2021**	Outcome of interest not assessed
**18**	**Baggus EMR. 2019**	Review article
**19**	**Lindo-Ricce 2017**	Outcome of interest not assessed
**20**	**Waheed N. 2016**	Outcome of interest not assessed
**21**	**Bernstein EF, 1988**	CD patients not included
**22**	**Rolny P. 1999**	CD patients not included
**23**	**Colcher H. 1955**	Outcome of interest not assessed
**24**	**Peters TJ. 1977**	Outcome of interest not assessed
**25**	**Sari S. 2016**	Outcome of interest not assessed
**26**	**Rai AA. 2015**	Outcome of interest not assessed
**27**	**Sanchez-Muñoz LB. 2008**	Outcome of interest not assessed
**28**	**Plane D. 1998**	Outcome of interest not assessed
